# Primary Carcinosarcoma of the Spleen: A Rare Case Report of Incidental Finding after Splenic Trauma

**DOI:** 10.1155/2020/8816931

**Published:** 2020-10-10

**Authors:** Hugues Ndasu Matendo, Raouf Fayisall Geraldo, Liviu Musteata, Joel Allan Green, Valeriu Krasovski, Valentin Nitu, Thierry Gastaud, Fabrice Cattan

**Affiliations:** ^1^Visceral Surgery Department, Moulins-Yzeure Hospital Centre (France), 10 Avenue du Général de Gaulle, 03006 Moulins, France; ^2^Clinique la Fraternité (Congo Kinshasa), 22 Avenue Uvira, Quartier Rombe I, Uvira, Congo; ^3^Health Sciences Faculty, University of Lome, Togo; ^4^West Michigan Surgical Specialists, 1045 Gezon Parkway SW, Wyoming, MI 49509, USA

## Abstract

Primary carcinosarcoma of the spleen is a rare, aggressive splenic malignancy. To date, seven cases have been reported in the literature. We report a first case of primary carcinosarcoma of the spleen in France. A 75-year-old woman with a medical history of hysterectomy for uterine adenocarcinoma presented with left hypochondrium pain following blunt abdominal trauma. A splenic mass was noted on computed tomography (CT) scan. A splenectomy was performed by laparotomy. Histology revealed a malignant mixed Mullerian tumor. The PET scan allowed us to confirm that it was a primary lesion of the spleen. She is currently undergoing adjuvant chemotherapy despite the tumor progression. The interest of this case lies in the rarity of primary carcinosarcomas of the spleen and the circumstances of its diagnosis.

## 1. Introduction

Carcinosarcomas are tumors composed of intimately mixed malignant epithelial and mesenchymal elements [1]. Most of carcinosarcoma appears in the genital tissue, and extragenital localizations are extremely rare [2]. They can develop at the expense of other organs, such as the peritoneum, salivary glands, lungs, esophagus, stomach, colon, pancreas, hepatobiliary system, thyroid, thymus, breast, urinary tract, and skin.

In the spleen, malignant tumors are most often of lymphoreticular origin [1, 3]. The discovery of carcinosarcoma in the spleen is extremely rare. To the best of our knowledge, there have been seven reported cases of primary spleen carcinosarcomas in the world literature (Table 1).

Westra et al. reported the first case of primary splenic carcinosarcoma, which is said to originate from the mesothelium, reflecting the unique capacity of the female mesothelium for Mullerian differentiation [4, 5]. Despite the rarity of this location, two male cases were presented by Rao et al. [6] and Kochar et al. [1] in England in the same health facility. These two cases share the same characteristics, namely, age, main symptomatology, and measurements on gross examination of the spleen. However, for the case of Rao et al., there is an absence of pathological history with an increase in the levels of human chorionic gonadotropin *β* (*β*-hCG). Joy et al. reported another case of primary splenic carcinosarcoma. However, no evidence of imaging, histology, or immunohistochemistry has been provided [5, 7]. Sun et al. [5] reported a case of splenic carcinosarcoma with the presence of local invasion of the chest wall. Ramson et al. [8] documented a first case which benefited from a laparoscopic splenectomy. More recently, Kwok [9] presented a case which underwent a splenectomy for primary carcinosarcoma of the spleen and in which an extragenital pelvic metastasis of the said carcinosarcoma was discovered during a second laparotomy.

Here, we report a case of primary carcinosarcoma of the spleen. The objectives are to describe the circumstances of the diagnosis and the care provided and to review the literature.

## 2. Case Presentation

A 75-year-old female patient was admitted for abdominal pain localized in the left hypochondrium. She had a history of blunt abdominal trauma 2 months earlier and a dry cough, which had developed for several weeks without any specific care. Physical examination revealed good general condition with overweight (BMI 26.93 kg/m^2^), a stable hemodynamic state, and a tenderness in the left hypochondrium but no palpable masses and lymph nodes.

Six years ago, she had undergone laparotomy for hysterectomy with bilateral oophorectomy with ilio-obturator dissection. The pathology revealed an endometrial adenocarcinoma T1a grade 1, with the recommendation of simple follow-up every 6 months for 2 years.

The patient also has past medical history of high blood pressure, herniated disc surgery, and atopy, including allergies to iodine, and intolerance to tramadol, codeine, and nefopam.

Blood analysis showed a slight anemia with hemoglobin at 11.2 g/dL and an elevation of platelets, LDH to 747 *μ*/L, and C-reactive protein raised to 155 mg/L. Tumor markers, including carcinoembryonic antigen (CEA), CA 19-9, and alpha-fetoprotein (AFP), as well as other hematological and biochemical parameters were normal.

**Figure 1:**
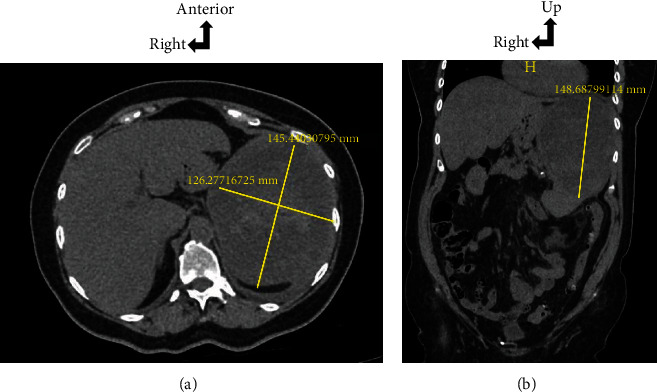
(a, b) Voluminous subcapsular hematoma of the upper pole of the spleen of 145 × 126 × 148 mm with low abundance intraperitoneal effusion ((a) axial section; (b) frontal reconstruction).

During her hospitalization, a thoracoabdominal CT (Figures 1(a) and 1(b)) without injection due to allergy to iodine was performed with evidence of thoracic bilateral pleural effusion and a suspected large hematoma under the spleen capsule of 145 × 126 mm with little free fluid in the abdominal cavity.

Due to the persistence of pain despite analgesic treatment (paracetamol and morphine) and uncertainty of diagnosis, we performed an exploratory laparotomy. This was done four days after admission through a midline laparotomy. A large spleen deformed by a probable large hematoma with extremely dense adhesions between this spleen and the prerenal fat as well as the diaphragm was found. There was no invasion of neighboring organs or locoregional lymphadenopathy; the other abdominal organs were normal in appearance. A splenectomy was performed.

The postoperative follow-up was simple with the prescription of Sandostatin, due to suspected pancreatic tail injury.

The patient was discharged on the seventh day after surgery with a low molecular weight heparin prescription for 3 weeks, as well as vaccines against *Streptococcus pneumoniae* and *Neisseria meningitidis* bacteria, *Haemophilus influenzae type B*, and *influenza* virus. She also received 2 million units of Oracillin daily for a period of 2 years.

Macroscopically, the spleen weighed 460 g and measured 20 × 14 × 8 cm. There was a capsule breach of 8 cm. On cutting, a voluminous nodular lesion, 12 cm in diameter, with a completely necrotic center was observed. At the periphery of this necrosis, there was a suspicious whitish tissue.

Histopathological examination revealed that the nodular lesion responsible for splenic rupture was a necrotic malignant neoplasm. In the tumor tissue studied, there were two distinct contingents: a tubulopapillary proliferation whose very basophilic coating included marked nuclear atypia and mitosis. These tumor glands were surrounded by atypical support tissue, made up of cells sometimes spindle shaped, sometimes large polyhedral with nuclear monstrosity.

Several immunohistochemical stains were performed: the epithelial components stained positively for cytokeratin 7 (CK7) and pancytokeratin AE1/AE3 while the sarcomatoid components stained positively for vimentin and CD10. The tumor was also positive for PAX8, WT1, and P16 and between 15 to 20% for KI67 but negative for p53, CD45, actin, desmin, and myogenin. The overall morphology and specific staining characteristics have made it possible to discuss the diagnosis of carcinosarcoma of Mullerian origin possibly secondary with suspicion of a primary tumor, either peritoneal or genital, given the history.

The PET scan, done postoperatively, showed FDG-avid areas around the splenectomy area, particularly at the level of the greater curvature of the stomach and the colonic region. This result led to a diagnosis of primary carcinosarcoma of the spleen with peritoneal involvement given the absence of other FDG-avid foci other than those around the splenectomy area.

Postoperatively, she was put on chemotherapy. Six months after surgery, because of the tumor progression, namely, a local recurrence with peritoneal carcinomatosis confirmed by CT and PET scan, after three cycles of Taxol carboplatin then two cycles of anthracycline (doxorubicin), the patient was put on Gemcitabine.

To date, 8 months after surgery, she is in her second cycle of Gemcitabine. In addition to the peritoneal carcinomatosis which slightly declined under this latter treatment, the patient did not present any other secondary lesions.

## 3. Discussion

Virchow, in 1864, was the first to use the term carcinosarcoma for the description of a tumor associating a carcinomatous component with a sarcomatous component [10]. These are neoplasms whose typical primary site is the female genital tract, with high metastatic potential [11]. The diagnosis is most often incidental. Both primary and secondary involvement of the spleen is rare [3]. To date, seven cases of primary carcinosarcomas of the spleen have been reported in the literature (Table 1).

Patient age ranged between 57 and 77 years, including four females who were postmenopausal.

The clinical presentation is not specific: in seven cases, five [1, 4–6, 9] had a painful mass in the left upper quadrant while the other two had anemia and painless mass of the upper left quadrant. In our case, it is a 75-year-old woman who was asymptomatic before the abdominal trauma. She presented with pain in the left hypochondrium after this trauma.

Histologically, our patient's spleen was the smallest in the series and weighed 460 g.

The carcinomatous component often combines a high-grade serous component, grade 3 endometrioide, with clear or undifferentiated cells. Depending on the sarcomatous component, two types are defined: either the sarcomatous component is normally present within the organ, we will then speak of homologous carcinosarcoma, or the component is formed from elements usually absent (cartilage tissue, bone, muscle fibers striated,…), we will then speak of heterologous carcinosarcoma (the most frequent form) [12].

The carcinogenesis and the biological behavior of carcinosarcoma are not fully understood.

Four theories have been put forward after comparing the epidemiological, clinical, histopathological, biological, and immunohistochemical profiles [5, 13, 14]. First, *the collision theory*, underpinned by a biclonal origin: two tumors (epithelial and mesenchymal) would emerge simultaneously from two separate stem cells. Second is *the composition theory*, which suggests that the mesenchymal tumor represents a pseudosarcomatous reaction of the epithelial tumor. Third, *the combination theory*, underpinned by a monoclonal origin: a common stem cell would alternately generate epithelial and mesenchymal cancer cells. Fourth, *the theory of conversion*, which also supports a monoclonal origin: from a stem cell would develop a tumor either epithelial or mesenchymal which would secondarily differentiate into the other tumor type, in particular an epithelial origin with a mesenchymal metaplasia.

Despite the remaining uncertainty about the mechanisms that generate these tumors, recent immunohistochemical, ultrastructural, and molecular genetic studies suggest and promote the concept of monoclonality in carcinosarcoma [13]. In addition, identical p53 and KRAS mutations have been identified in the epithelial and mesenchymal components of carcinosarcoma, results which suggest an early alteration in the histogenesis of the tumor with late transformation or degeneration of the epithelial component into the sarcomatous component [13].

However, the immunohistochemical study has little interest apart from the demonstration of a heterologous contingent; in other words, it is not essential if the histological aspect is characteristic. The epithelial components are generally positive for cytokeratin, while the sarcomatous tissue is generally positive for vimentin [4, 9], which is consistent with the staining pattern in our case.

To date, there is no consensus for the treatment of splenic carcinosarcoma. It is generally accepted that early surgery with complete macroscopic resection (splenectomy) of the malignant tumor may offer the best chance of survival [1, 5, 9].

However, one of the cases benefited from two surgeries for the same disease [9]: first, a total subcostal splenectomy; this did not allow the pelvis to be explored at the same time, with immediate postoperative discovery of a pelvic peritoneal metastasis that required a second laparotomy. One should consider the approaches that allow good exploration and management of a splenic mass:

- Laparoscopy, if possible, or robotic surgery with [8] or without a mini laparotomy to extract the spleen
- Exploratory laparotomy by the subcostal approach in the absence of other pathology
- Splenectomy by a midline incision

Postoperatively, adjuvant treatments, such as chemotherapy and radiotherapy, could be considered to improve the prognosis of patients [5]. For chemotherapy, both carcinomatous and sarcomatous components must be treated. Numerous chemotherapy regimens including ifosfamide, paclitaxel, cisplatin, and carboplatin have been tested, with no solid evidence indicating the superiority of one combination over another. However, it has been shown that the use of chemotherapy as single therapy results in lower overall survival [9, 15]. Postoperative irradiation has been shown to improve local control without benefit to overall survival. Multimodal therapy should lead to better results. Recently, there are many ongoing studies with molecular and targeted therapies to improve efficacy; however, relevant results may not be available for several years due to the small number of patients [11].

The prognosis for carcinosarcoma is poor, with splenic localization being no exception [8]. Postoperative survival is estimated between 3 and 12 months [1, 4–9].

## 4. Conclusion

Primary carcinosarcoma of the spleen is a rare entity with a poor prognosis. Very few cases have been reported in the literature. Two histological types are described: the heterologous type and the homologous type, but without affecting the prognosis. There is no consensus for adjuvant therapy with chemotherapy, radiotherapy, or targeted therapy. The surgical management should include splenectomy with complete exploration of the abdominal cavity.

## Figures and Tables

**Table 1:** Cases of primary carcinosarcoma of the spleen reported in the literature.

Case	Author (year) (ref)	Country	Age (years)	Sex	Weight (g) (spleen)	Distant metastasis	Outcome
1	Westra et al. (1994) [4]	United States	57	F	1680	FU	DD
2	Rao et al. (2007) [6]	England	60	M	2330	FU	DD
3	Kochar et al. (2009) [1]	England	60	M	2330	FU	DD
4	Joy et al. (2012) [7]	England	74	F	NP	IP	AWM
5	Sun et al. (2017) [5]	China	64	F	2980	IP	AWM
6	Ramson et al. (2019) [8]	Australia	77	M	895	FU	AWM
7	Kwok (2019) [9]	Australia	74	F	NP	IP	DD
8	Present case (2020)	France	75	F	460	No	AWM

IP: metastases detected at the initial presentation; FU: metastases detected during follow-up; D: died of disease; AWM: alive with the disease (at the time of publication); NP: not specified.

## Abbreviations

AE1/AE3: Cytokeratin (or pancytokeratin) AE1/AE3: keratin cocktail that detects CK1-8, 10, 14-16, and 19, but does not detect CK17 or CK18
CA: Cancer antigen
CD10: Cluster of differentiation 10
CD45: Cluster of differentiation 45
CEA: Carcinoembryonic antigen
CK7: Cytokeratin 7
KI67: A cellular marker for proliferation
KRAS: Kirsten rat sarcoma viral oncogene homolog
LDH: Lactate dehydrogenase
P16: (Also known as p16INK4a, cyclin-dependent kinase inhibitor 2A, CDKN2A, multiple tumor suppressor 1) tumor suppressor protein
p53: Tumor protein 53
PAX8: Paired box gene 8 (for primary or secondary Mullerian tumor)
PET: Positron emission tomography
WT1: Wilms tumor 1.
